# Supplementary material for: Modeling glioblastoma heterogeneity as a dynamic network of cell states
Source: Mol Syst Biol. 2021 Sep 16;17(9):e10105. doi: 10.15252/msb.202010105 (PMC8444284; doi:10.15252/msb.202010105)
Supplement: Supplementary file 6 — Source Data for Figure 5 [file MSB-17-e10105-s004.zip › Figure5A_sourcedata/GSEA_3071/hallmarks_stateIV.GseaPreranked.1623742551702/HALLMARK_EPITHELIAL_MESENCHYMAL_TRANSITION.html]

Details for gene set HALLMARK\_EPITHELIAL\_MESENCHYMAL\_TRANSITION[GSEA]

|  || Dataset | state53071 |
| Phenotype | NoPhenotypeAvailable |
| Upregulated in class | na\_neg |
| GeneSet | HALLMARK\_EPITHELIAL\_MESENCHYMAL\_TRANSITION |
| Enrichment Score (ES) | -0.60595125 |
| Normalized Enrichment Score (NES) | -3.3761106 |
| Nominal p-value | 0.0 |
| FDR q-value | 0.0 |
| FWER p-Value | 0.0 |
Table: GSEA Results Summary

  

Fig 1: Enrichment plot: HALLMARK\_EPITHELIAL\_MESENCHYMAL\_TRANSITION      
 Profile of the Running ES Score & Positions of GeneSet Members on the Rank Ordered List

  

| PROBE | GENE SYMBOL | GENE\_TITLE | RANK IN GENE LIST | RANK METRIC SCORE | RUNNING ES | CORE ENRICHMENT || 1 | IGFBP2 |  |  | 16 | 2.364 | 0.0120 | No |
| 2 | CADM1 |  |  | 38 | 1.965 | 0.0205 | No |
| 3 | IGFBP3 |  |  | 59 | 1.794 | 0.0281 | No |
| 4 | TNC |  |  | 66 | 1.751 | 0.0381 | No |
| 5 | CDH2 |  |  | 68 | 1.735 | 0.0490 | No |
| 6 | DPYSL3 |  |  | 75 | 1.691 | 0.0586 | No |
| 7 | COL1A2 |  |  | 137 | 1.425 | 0.0559 | No |
| 8 | ID2 |  |  | 149 | 1.378 | 0.0626 | No |
| 9 | FBLN1 |  |  | 213 | 1.226 | 0.0582 | No |
| 10 | PFN2 |  |  | 270 | 1.107 | 0.0544 | No |
| 11 | SCG2 |  |  | 294 | 1.040 | 0.0566 | No |
| 12 | FZD8 |  |  | 325 | 0.998 | 0.0572 | No |
| 13 | PMEPA1 |  |  | 373 | 0.939 | 0.0540 | No |
| 14 | PDLIM4 |  |  | 435 | 0.867 | 0.0478 | No |
| 15 | FBN2 |  |  | 534 | 0.781 | 0.0338 | No |
| 16 | PRRX1 |  |  | 653 | 0.689 | 0.0153 | No |
| 17 | GAS1 |  |  | 807 | 0.600 | -0.0105 | No |
| 18 | SAT1 |  |  | 859 | 0.575 | -0.0167 | No |
| 19 | VEGFA |  |  | 1010 | 0.518 | -0.0425 | No |
| 20 | APLP1 |  |  | 1035 | 0.510 | -0.0439 | No |
| 21 | EFEMP2 |  |  | 1311 | 0.438 | -0.0944 | No |
| 22 | SLC6A8 |  |  | 1365 | 0.424 | -0.1020 | No |
| 23 | CAP2 |  |  | 1410 | 0.414 | -0.1079 | No |
| 24 | HTRA1 |  |  | 1445 | 0.404 | -0.1119 | No |
| 25 | SGCB |  |  | 1593 | 0.369 | -0.1380 | No |
| 26 | CXCL12 |  |  | 1788 | 0.333 | -0.1735 | No |
| 27 | CDH6 |  |  | 1920 | 0.310 | -0.1969 | No |
| 28 | SFRP1 |  |  | 1979 | 0.301 | -0.2062 | No |
| 29 | MSX1 |  |  | 2021 | 0.293 | -0.2123 | No |
| 30 | CD44 |  |  | 2197 | 0.268 | -0.2445 | No |
| 31 | ABI3BP |  |  | 2223 | 0.265 | -0.2477 | No |
| 32 | PPIB |  |  | 2412 | -0.259 | -0.2824 | No |
| 33 | PVR |  |  | 2496 | -0.269 | -0.2968 | No |
| 34 | MYLK |  |  | 2532 | -0.273 | -0.3019 | No |
| 35 | FGF2 |  |  | 2613 | -0.284 | -0.3156 | No |
| 36 | RHOB |  |  | 2638 | -0.288 | -0.3184 | No |
| 37 | PTHLH |  |  | 2731 | -0.297 | -0.3343 | No |
| 38 | ECM2 |  |  | 2780 | -0.303 | -0.3417 | No |
| 39 | JUN |  |  | 2785 | -0.304 | -0.3405 | No |
| 40 | ITGB1 |  |  | 2817 | -0.308 | -0.3445 | No |
| 41 | GPX7 |  |  | 2856 | -0.314 | -0.3499 | No |
| 42 | TNFRSF12A |  |  | 2890 | -0.318 | -0.3543 | No |
| 43 | ITGB3 |  |  | 2905 | -0.319 | -0.3549 | No |
| 44 | LAMA2 |  |  | 3235 | -0.366 | -0.4164 | No |
| 45 | TNFAIP3 |  |  | 3248 | -0.368 | -0.4164 | No |
| 46 | PLOD2 |  |  | 3453 | -0.400 | -0.4533 | No |
| 47 | COL8A2 |  |  | 3596 | -0.428 | -0.4781 | No |
| 48 | GEM |  |  | 3609 | -0.430 | -0.4777 | No |
| 49 | LGALS1 |  |  | 3692 | -0.443 | -0.4908 | No |
| 50 | TPM4 |  |  | 3761 | -0.458 | -0.5010 | No |
| 51 | CALD1 |  |  | 3763 | -0.458 | -0.4983 | No |
| 52 | NOTCH2 |  |  | 3853 | -0.477 | -0.5125 | No |
| 53 | ITGA5 |  |  | 3864 | -0.480 | -0.5114 | No |
| 54 | FERMT2 |  |  | 4151 | -0.555 | -0.5633 | No |
| 55 | IL15 |  |  | 4186 | -0.564 | -0.5663 | No |
| 56 | LOXL1 |  |  | 4206 | -0.569 | -0.5663 | No |
| 57 | VIM |  |  | 4255 | -0.582 | -0.5719 | No |
| 58 | ITGAV |  |  | 4312 | -0.601 | -0.5789 | No |
| 59 | GADD45A |  |  | 4373 | -0.626 | -0.5865 | No |
| 60 | SDC4 |  |  | 4412 | -0.641 | -0.5898 | No |
| 61 | FSTL3 |  |  | 4423 | -0.643 | -0.5876 | No |
| 62 | FSTL1 |  |  | 4446 | -0.653 | -0.5877 | No |
| 63 | VCAN |  |  | 4464 | -0.661 | -0.5868 | No |
| 64 | QSOX1 |  |  | 4559 | -0.712 | -0.6005 | No |
| 65 | BMP1 |  |  | 4563 | -0.713 | -0.5965 | No |
| 66 | THY1 |  |  | 4587 | -0.729 | -0.5963 | No |
| 67 | NT5E |  |  | 4607 | -0.737 | -0.5953 | No |
| 68 | SDC1 |  |  | 4663 | -0.770 | -0.6010 | Yes |
| 69 | PLOD1 |  |  | 4685 | -0.788 | -0.6001 | Yes |
| 70 | P3H1 |  |  | 4688 | -0.788 | -0.5954 | Yes |
| 71 | COL5A3 |  |  | 4689 | -0.789 | -0.5904 | Yes |
| 72 | MCM7 |  |  | 4704 | -0.800 | -0.5880 | Yes |
| 73 | TGFB1 |  |  | 4781 | -0.846 | -0.5973 | Yes |
| 74 | CAPG |  |  | 4812 | -0.877 | -0.5975 | Yes |
| 75 | LOXL2 |  |  | 4816 | -0.881 | -0.5925 | Yes |
| 76 | BDNF |  |  | 4818 | -0.882 | -0.5870 | Yes |
| 77 | MGP |  |  | 4824 | -0.890 | -0.5823 | Yes |
| 78 | DKK1 |  |  | 4828 | -0.893 | -0.5772 | Yes |
| 79 | SERPINH1 |  |  | 4829 | -0.895 | -0.5715 | Yes |
| 80 | TGM2 |  |  | 4837 | -0.903 | -0.5671 | Yes |
| 81 | MATN3 |  |  | 4943 | -1.005 | -0.5810 | Yes |
| 82 | EMP3 |  |  | 4960 | -1.029 | -0.5775 | Yes |
| 83 | FLNA |  |  | 4975 | -1.051 | -0.5735 | Yes |
| 84 | MMP14 |  |  | 4983 | -1.064 | -0.5681 | Yes |
| 85 | IL32 |  |  | 5021 | -1.128 | -0.5680 | Yes |
| 86 | NNMT |  |  | 5039 | -1.168 | -0.5639 | Yes |
| 87 | NTM |  |  | 5052 | -1.188 | -0.5586 | Yes |
| 88 | MFAP5 |  |  | 5068 | -1.222 | -0.5537 | Yes |
| 89 | TIMP1 |  |  | 5086 | -1.257 | -0.5490 | Yes |
| 90 | COL7A1 |  |  | 5088 | -1.260 | -0.5411 | Yes |
| 91 | GLIPR1 |  |  | 5096 | -1.279 | -0.5343 | Yes |
| 92 | COL5A2 |  |  | 5097 | -1.282 | -0.5261 | Yes |
| 93 | GADD45B |  |  | 5099 | -1.292 | -0.5181 | Yes |
| 94 | TGFBI |  |  | 5104 | -1.305 | -0.5105 | Yes |
| 95 | VEGFC |  |  | 5113 | -1.319 | -0.5036 | Yes |
| 96 | LOX |  |  | 5149 | -1.418 | -0.5013 | Yes |
| 97 | LRP1 |  |  | 5152 | -1.437 | -0.4925 | Yes |
| 98 | BGN |  |  | 5160 | -1.486 | -0.4844 | Yes |
| 99 | SERPINE1 |  |  | 5162 | -1.501 | -0.4750 | Yes |
| 100 | COL11A1 |  |  | 5169 | -1.526 | -0.4664 | Yes |
| 101 | MATN2 |  |  | 5172 | -1.530 | -0.4570 | Yes |
| 102 | COL12A1 |  |  | 5194 | -1.634 | -0.4507 | Yes |
| 103 | BASP1 |  |  | 5203 | -1.665 | -0.4416 | Yes |
| 104 | IGFBP4 |  |  | 5211 | -1.701 | -0.4321 | Yes |
| 105 | PLAUR |  |  | 5213 | -1.721 | -0.4213 | Yes |
| 106 | CDH11 |  |  | 5218 | -1.759 | -0.4108 | Yes |
| 107 | THBS1 |  |  | 5222 | -1.800 | -0.3999 | Yes |
| 108 | ITGA2 |  |  | 5234 | -1.908 | -0.3898 | Yes |
| 109 | COL4A2 |  |  | 5235 | -1.917 | -0.3776 | Yes |
| 110 | DCN |  |  | 5237 | -1.928 | -0.3655 | Yes |
| 111 | SNAI2 |  |  | 5238 | -1.940 | -0.3531 | Yes |
| 112 | TPM2 |  |  | 5242 | -1.971 | -0.3411 | Yes |
| 113 | PCOLCE |  |  | 5247 | -2.050 | -0.3287 | Yes |
| 114 | DAB2 |  |  | 5250 | -2.114 | -0.3156 | Yes |
| 115 | PDGFRB |  |  | 5253 | -2.141 | -0.3023 | Yes |
| 116 | TPM1 |  |  | 5254 | -2.143 | -0.2886 | Yes |
| 117 | COL6A2 |  |  | 5258 | -2.256 | -0.2748 | Yes |
| 118 | PTX3 |  |  | 5262 | -2.327 | -0.2605 | Yes |
| 119 | FBN1 |  |  | 5263 | -2.334 | -0.2456 | Yes |
| 120 | CTHRC1 |  |  | 5265 | -2.365 | -0.2307 | Yes |
| 121 | COL4A1 |  |  | 5270 | -2.478 | -0.2156 | Yes |
| 122 | LUM |  |  | 5271 | -2.487 | -0.1998 | Yes |
| 123 | MYL9 |  |  | 5272 | -2.509 | -0.1837 | Yes |
| 124 | COL5A1 |  |  | 5276 | -2.605 | -0.1677 | Yes |
| 125 | SPP1 |  |  | 5277 | -2.671 | -0.1506 | Yes |
| 126 | INHBA |  |  | 5280 | -2.740 | -0.1335 | Yes |
| 127 | EDIL3 |  |  | 5282 | -2.918 | -0.1150 | Yes |
| 128 | FN1 |  |  | 5284 | -3.017 | -0.0960 | Yes |
| 129 | TAGLN |  |  | 5285 | -3.021 | -0.0767 | Yes |
| 130 | COL3A1 |  |  | 5287 | -3.508 | -0.0544 | Yes |
| 131 | COL6A3 |  |  | 5288 | -3.654 | -0.0311 | Yes |
| 132 | COL1A1 |  |  | 5290 | -4.897 | -0.0000 | Yes |
Table: GSEA details [plain text format]

  

Fig 2: HALLMARK\_EPITHELIAL\_MESENCHYMAL\_TRANSITION: Random ES distribution      
 Gene set null distribution of ES for **HALLMARK\_EPITHELIAL\_MESENCHYMAL\_TRANSITION**
